# Diabetes Specialist Nurses’ Experiences of Supporting Emerging Adults Living With Type 1 Diabetes Mellitus After the Transfer to Adult Care—A Qualitative Study

**DOI:** 10.1155/nrp/4036033

**Published:** 2025-12-20

**Authors:** S. Olsson, T. Willman, Å. Hörnsten, J. Otten, M. Blusi, E. Lundberg, I. Lavin, Å. Carlsund

**Affiliations:** ^1^ Department of Nursing, Umeå University, Umeå, Sweden, umu.se; ^2^ North Älvsborgs Hospital, Paediatric Diabetes Clinic, Trollhättan, Sweden; ^3^ Department of Public Health and Clinical Medicine, Umeå University, Umeå, Sweden, umu.se; ^4^ Department of Paediatrics, Institution of Clinical Sciences, Umeå University, Umeå, Sweden, umu.se; ^5^ Umeå University Hospital, Paediatric Diabetes Clinic, Umeå, Sweden, umu.se

## Abstract

**Aim:**

To explore and describe the experiences of Swedish diabetes specialist nurses in supporting emerging adults with type 1 diabetes mellitus after the transfer from paediatric to adult care.

**Background:**

The transfer from paediatric to adult care for emerging adults living with type 1 diabetes mellitus is a critical period that can impact their well‐being and long‐term health outcomes. Diabetes specialist nurses play a crucial role in supporting these individuals during the transitions in emerging adulthood. However, their experiences and challenges in providing optimal support have not been extensively studied.

**Method:**

We used a qualitative descriptive design. Ten diabetes nurse specialists from six different Swedish hospitals participated in recorded individual interviews by video link or face‐to‐face using a semistructured interview guide. Data collection spanned from May to December 2023, and the data were analysed with qualitative content analysis. Informed consent was obtained.

**Results:**

The main theme of the results was ambition to optimise individual support but with hands tied by structural barriers with three additional themes concentrating on the individual and the relationship, emphasising long‐term objectives in self‐management support, and struggling with obstacles to providing accessible care. The results indicate that while diabetes specialist nurses focus on building strong relationships and emphasise long‐term goals in self‐management support, they encounter significant obstacles when providing accessible care after the transfer to adult care.

**Conclusion:**

Diabetes specialist nurses highlighted a need for education and training that extends beyond simply managing the medical condition, considering various life transitions and psychosocial challenges. The findings indicate that future standards for supportive interventions should prioritise existential and psychosocial factors. There is a lack of systematic national consensus on collaboration, as the diabetes specialist nurses reported differing experiences regarding transfers and continuity of care.

## 1. Introduction

Several lifelong illnesses, such as type 1 diabetes mellitus (T1DM), mainly have their onset in childhood or adolescence, leading to rising numbers of individuals being transferred to adult care [1]. Sweden has the world’s second‐highest incidence of T1DM in children and adolescents [2], leading to particularly large expected numbers of emerging adults (EAs) transferring to adult care in this country. Self‐management while living with T1DM includes, for instance, taking medication, monitoring your health, eating healthily, staying active, reducing risks, and solving problems [3]. Parental involvement in T1DM self‐management during adolescence is linked to more optimal treatment outcomes, but the parental role naturally becomes less intensive as the adolescents’ independence increases [4, 5]. Emerging adulthood (ages 18–29) is a life stage characterised by identity exploration, changes in residence, self‐focus, transitions marked by feelings of liminality, and optimism [6]. Living in adolescence and emerging adulthood involves complex challenges concerning biological and psychosocial needs, and T1DM adds further vulnerability [2, 4, 5, 7, 8].

Living with T1DM is demanding, even with access to supportive technology for self‐management, support, education, and diabetes care from healthcare professionals (HCPs) [2]. HCPs in the diabetes team must recognise shifts in individual conditions during life transitions, including changes in attitudes, beliefs, knowledge, expectations, and social roles [9]. Standard of care strives for optimal treatment outcomes and psychosocial well‐being [4, 8]. The demands of emerging adulthood and the increasing number of EAs living with T1DM transferring to adult care may redefine quality indicators for assessing health outcomes and care for this group [1].

In Sweden, the transfer from paediatric to adult care typically occurs at 18, marking the beginning of emerging adulthood. Self‐management is frequently linked to practical interventions, but T1DM treatment should adopt a holistic approach that includes psychological support [4, 10]. Diabetes specialist nurses (DSNs) play central roles in the diabetes team, supporting EAs [11]. The DSNs are expected to communicate and connect with patients, identify challenges, conduct psychosocial screenings, evaluate, and adapt their nursing interventions to the unique context of EAs [4, 8]. However, they often face challenges due to a lack of resources to address the psychosocial needs of EAs [7], even in paediatric care [12].

No Swedish guidelines cover routines for transferring EAs to adult care, but international guidelines stress the importance of support from DSNs after transfer. Studies, such as the STEPSTONES‐DIAB project, focus on preparing adolescents for this transfer by encouraging them to take an active role. Despite several interventions aimed at preparing adolescents for the transfer, further interventions in adult care are necessary to ensure ongoing support [13]. The transfer from paediatric to adult care is a high‐risk period for EAs, often leading to interruptions in their health and care [14]. Stressing the importance of this transfer for DSNs’ experiences of supporting EAs, this study aims to describe DSNs’ experiences of supporting EAs living with T1DM after the transfer from paediatric to adult care.

## 2. Method

### 2.1. Design

We used a qualitative descriptive design to explore DSNs’ experiences of supporting EAs living with T1DM after the transfer from paediatric to adult care. We used a qualitative descriptive design to give voice to DSNs by exploring and describing their variations in experiences. In addition, we chose this design to contribute to the utilisation of research results in nursing practice.

### 2.2. Researcher Positionality

The disciplinary roles of this research team were PhD‐student (SO), Associate Professor (ÅC, JO, MB), PhD (EL), and Professor (ÅH). The professional roles of this research team were paediatric nurse specialist (SO, TW, and IL), community health nurse (ÅH, ÅC), diabetes nurse specialist (IL), registered nurse (MB), endocrinologist (JO), and paediatric endocrinologist (EL). All research team members were female. Senior research members mentored the members who were developing their skills in qualitative research. The interviewers (TW and SO) had no prior relationships with the participants. The first interviewer (TW) had experience in working with diabetes in paediatric care supporting adolescents and parents before transferring to adult care. The second interviewer (SO) had experience in living with T1DM and education in diabetes care. The preunderstanding was discussed continuously during the process of qualitative content analysis. The impacts of preunderstanding are discussed in 4.1 Trustworthiness.

### 2.3. Sampling and Recruitment

Emails seeking recruitment approval were sent to 12 department heads overseeing adult diabetes teams at 12 Swedish hospitals. Once approved, information emails were sent to the DSNs. We (SO and TW) had correspondence with potential participants, and the DSNs were able to ask questions before signing informed consent forms. The interviewers (SO and TW) scheduled the interviews with respect to the participants’ work schedules and if they preferred an interview face‐to‐face instead of by video link. A purposive sampling was used, meaning that we recruited DSNs who fulfilled the inclusion criteria of having minimum 1 year of work experience in supporting EAs living with T1DM in adult diabetes care. Ten DSNs accepted to participate in individual interviews. The participants ranged in age from 29 to 62 years (median age 45), were female, and had one to 18 years of work experience in diabetes care (median 11 years). The participants worked in adult diabetes teams at specialized care clinics at seven different hospitals, geographically spread across Sweden. The DSNs worked in adult diabetes teams ranging from supporting approximately 70–190 EAs living with T1DM, along with other patients living with diabetes. In the Swedish setting, the DSN has a central role caring for EAs with regular communication and office visits as compared to physicians, who in general have contact with EAs once a year.

### 2.4. Data Collection

Data collection spanned from May to December 2023. Ten individual semistructured interviews were conducted. The individual interviews utilised a semistructured interview guide. The interview guide emphasised topics such as the pros and cons during the transitions and subjects of improvement. A structured pilot testing of the interview guide was not performed, but the guide was considered to serve its purpose during the first interview and was thereby used for the subsequent interviews. It was developed to ensure consistency in the questions asked and still permit further exploration of topics of interest. The interview guide was also developed to ensure consistency in data collection since we had two separate interviewers with different experiences conducting interviews (TW and SO). We decided to provide the participants with brief information about the interviewers, the preunderstanding, and the aim of the study as an introduction. The two interviewers (TW and SO) performed the interviews and transcripts separately. The interviews were conducted either by video link (*n* = 6) or face‐to‐face at the DSNs’ workplaces (*n* = 4). Audio and visual recording was performed. The interviews lasted 16–52 (median 31) minutes and were transcribed verbatim.

### 2.5. Data Analysis

The transcribed interview data were analysed using the analytic method of qualitative content analysis according to Graneheim and Lundman [15] and Graneheim et al. [16]. The method focuses on describing a variation of content on various levels of abstraction and interpretation [17]. The process started with reading the transcripts multiple times. Thereby, an overview was obtained. Thereafter, the transcribed text was analysed line by line, starting with identifying meaning units corresponding to the aim (SO and TW). During the decontextualisation phase, these meaning units were condensed based on their content, i.e., being shortened and given labels/codes without losing the core meaning (SO and TW both individually and paired). During the recontextualisation phase, the codes were organised into groups based on similarities and differences. The recontextualisation phase included several video link consensus meetings where disagreements were discussed until agreement (SO and TW). These groups of codes that were given preliminary labels were later scrutinised to identify levels of abstraction and interpretation, whereby a preliminary overview of subthemes and themes was discussed to reach consensus in video link gatherings (SO, TW, ÅH, and ÅC). We decided to end the data collection when the ninth and tenth interviews did not reveal any new emerging subthemes. The final overview with themes and subthemes was discussed among all research team members and thereby somewhat adjusted in agreement. Ultimately, a main theme was identified covering all the data. The qualitative content analysis process is not linear, as the method requires alternating between reading the transcribed texts, sorting codes, interpreting, and abstracting at various levels [15, 17]. The main analytical process was conducted in Swedish. The transfer to the English language started when the final overview was agreed upon and the writing of the Results started. To ensure the results accurately reflected the original meanings, we continuously reviewed and discussed the language aspects.

### 2.6. Ethical Considerations

The study was conducted in accordance with ethical standards by the World Medical Association’s Declaration of Helsinki [18], and written informed consent was obtained from participants and their department heads. Before the interviews, participants were informed about the study’s purpose, risks, benefits, and their voluntary participation in the study. Results are presented confidentially to protect their integrity and identities. Descriptions of the participants′ characteristics, as well as the data collection and analysis methods, were included to support the transferability of the findings. Including quotations with the findings enables readers to assess the applicability of the results to different settings. To prevent unauthorised access, the data were processed in accordance with the General Data Protection Regulation (GDPR 2016/679). Each interview was labelled with a number, and the participants’ informed consent was obtained. The interviews were stored in a secure data collection tool at Umeå University, where only the researchers responsible for the study had access. An ethical approval (Dnr 2023‐03027‐01) was obtained from the Swedish Ethical Review Authority.

## 3. Results

The 10 Swedish DSNs aimed to optimise support for EAs but faced organisational barriers. They focused on individual relationships and long‐term self‐management goals yet encountered challenges in providing accessible care after the transfer from paediatric to adult care. Main results are summarised in Table 1, with themes and quotations presented in the text.

**Table 1:** The results: main theme, themes, and subthemes.

Main theme	Themes	Subthemes
Ambition to optimise individual support but with hands tied by structural barriers	Concentrating on the individual and the relationship	Exploring the person behind the disease Building a trusting caring relationship Adapting care to individual needs Involving significant others wisely
	Emphasising long‐term objectives in self‐management support	Showing patience and understanding Instilling hope for the future Supporting gradual independence
	Struggling with obstacles to providing accessible care	Managing the distance from paediatric care Dealing with organisational challenges Attempting to implement updated contact pathways

### 3.1. Concentrating on the Individual and the Relationship

The DSNs concentrated on the individuals and their relationships with them when supporting EAs living with T1DM after the transfer. They explored the individual behind the disease, tried to build a personal caring relationship, adapted the care to individual needs, and strove to involve significant others wisely.

#### 3.1.1. Exploring the Person Behind the Disease

The DSNs emphasised the need to understand the person behind the disease during the initial visits. They aimed to learn about the EA living with T1DM by asking about their interests, challenges, and healthcare experiences. A DSN emphasised the importance of understanding the EA’s perspective on living with the constant presence of the illness:

I ask what kind of relationship they have with their diabetes. About everything, how it has been over the years and how they have experienced living with an illness like this. I believe this says a lot. (Participant 1)

#### 3.1.2. Building a Trusting Caring Relationship

The DSNs aimed to build trusting, caring relationships and created a welcoming atmosphere by sharing personal details and making meetings more relaxed, focusing on encouraging open discussions about sensitive topics and patiently waiting for the EAs to engage. However, they faced challenges when EAs did not communicate:

We don’t want people [i.e., EAs] to be afraid to come here. Some people don’t show up at office visits and we don’t know why. I just want them to come and realise that I’m not dangerous. Just show up, we don’t have any requirements for them, but it’s hard to convey that when we haven’t met them and they’ve never been here. (Participant 2)

The DSNs worked intensely to build trusting relationships, understanding their importance for the EAs’ future care. Frequent visits during the first year were crucial. While the DSNs recognised the power dynamics, they did not seek a higher position, acknowledging it stemmed from care norms.

#### 3.1.3. Adapting Care to Individual Needs

The DSNs adapted care to individual needs, emphasising person‐centred care through thorough evaluations. They gathered information to adapt in meetings with EAs, beginning with a disease knowledge assessment to identify individuals’ prerequisites, knowledge levels, needs, and personal goals. DSNs differed in their preparation for initial meetings with EAs; some carefully reviewed medical records, while others preferred to approach the conversation without preconceptions:

I think it’s good to look at them with neutral eyes […]. Sometimes there have been previous difficulties, and I believe it’s not always good to know too much about them. I believe that they [i.e., the EAs] sense during the visits that we view them as clean slates. (Participant 7)

#### 3.1.4. Involving Significant Others Wisely

The DSNs identified significant others, primarily parents, and sought to involve them thoughtfully. They were supportive of parental participation during office visits, while always prioritising the confidentiality and integrity of the EA. Some DSNs noted a shift in perspective, now viewing parental involvement as natural and beneficial, in contrast to the stringent expectation that EAs should act as independent adults. Some HCPs felt that the DSNs were overprotective when it came to involving parents. During parental involvement, they often preferred to speak with the EA separately because, while family members can provide essential support, they may sometimes be seen as hindrances:

Sometimes when they [i.e., the EAs] visit together with a parent […] you can determine how open they are with each other. […] but I don’t know how open I can be when a parent is present. […] There are challenges, and I believe that many [EAs] want to keep up appearances with everything being perceived as fine. (Participant 9)

The DSNs worked to gain parents’ trust, acknowledging their concerns as emotionally challenging. They noted it was difficult when parents struggled to “let go,” potentially affecting the transfer and support. The DSNs encouraged EAs to bring a friend or partner to office visits to address social needs.

### 3.2. Emphasising Long‐Term Objectives in Self‐Management Support

The DSNs emphasised long‐term self‐management objectives when supporting EAs living with T1DM. They showed patience and understanding given the challenges encountered during this turbulent period, instilled hope for the future, and facilitated the EAs’ process of gradually gaining independence.

#### 3.2.1. Showing Patience and Understanding

The DSNs expressed a need to show patience and understanding given the burden of living with T1DM along with the multiple life transitions during emerging adulthood. It was challenging to show patience and still motivate EAs to achieve better treatment outcomes. They mentioned that EAs sometimes experienced burnout and stressed the importance of not blaming the EAs when experiencing suboptimal treatment outcomes:

Always motivate them. They should walk out of here [i.e., the clinic] and be able to feel like they are doing their best, even if they have an HbA1c value of 104. […] I say that living with diabetes is burdensome. I always say that I know that they don’t want to have these high blood glucose values […] of course they don’t, I see that. (Participant 6)

Emerging adulthood was described as a turbulent period of life, often linked to less optimal treatment adherence. The DSNs did their best to avoid lecturing and did not impose overly strict requirements but felt the urge to talk about what was suboptimal.

#### 3.2.2. Instilling Hope for the Future

The DSNs instilled hope for the future by helping motivate the EAs to strive for better treatment outcomes and well‐being, which is considered important but difficult. All improvements were acknowledged, even the smallest ones. The DSNs provided support and encouragement by addressing positive factors:

You can always find a day when everything is fine. And we keep the attention on what’s positive, while the patients are usually more aware of and notice what’s negative. (Participant 3)

EAs communicated openly, sharing dissatisfaction when necessary. DSNs felt engaged when connecting with EAs and exchanging knowledge. Some EAs struggled with their illnesses, affecting DSNs, especially when outreach efforts faltered, causing worry and feelings of failure. Personal factors like family planning often inspire hope in EAs, motivating them to improve treatment outcomes, with maturity and motivation generally increasing with age.

#### 3.2.3. Supporting Gradual Independence

The DSNs supported EAs in gaining independence, which varied based on factors like age at T1DM onset, early independence training, parental support, and personal maturity. Moving out of the parents’ home marked a key transition, with smoother transfers for those perceived as more independent. Continuous support for independence was vital, especially after the transfer to adult care:

I make sure to communicate that I see and hear them, and I reflect on what they tell me and ask if I’ve got it right. I act as a coach […] I want them to feel like they’re capable on their own. […] I want them to feel like it’s their own decisions. I think that’s great, because it further leads them to dare to believe in themselves. (Participant 8)

With the EAs’ growing age, the DSNs later emphasised ongoing support, coaching through education, identifying causal relationships, and teaching EAs how to identify these relationships in self‐management interventions. This helped the EAs better understand how to maintain and strive for optimal blood glucose levels in everyday life.

### 3.3. Struggling With Obstacles to Providing Accessible Care

The DSNs struggled with obstacles to providing accessible care when supporting EAs living with T1DM after the transfer to adult care. They managed the perceived distance from paediatric care, addressed organisational challenges, and identified a need to implement updated contact pathways.

#### 3.3.1. Managing Distance From Paediatric Care

The perceived distance from paediatric care needed to be managed. The DSNs described not always being able to meet families’ expectations of adult care, after the families were recently transferred, leaving behind trusted long‐term caring relationships in paediatric care:

It’s challenging to create trust and build a caring relationship […] but we have different roles versus at the paediatric clinic. It’s like starting uphill. (Participant 10)

The DSNs aimed to enhance collaboration with paediatric care to better prepare patients for transfer. Strategies included receiving written evaluations from EAs and early training in diabetes self‐management. Some clinics held joint transfer meetings, though opportunities were limited. DSNs occasionally invited paediatric HCPs to initial adult‐care visits with EA consent, primarily reserved for special cases, such as mental health issues. DSNs felt that some EAs were too exposed during these meetings and expressed discomfort during the final visits with paediatric HCPs.

#### 3.3.2. Dealing With Organisational Challenges

The DSNs highlighted a struggle to deal with organisational challenges, they had more patients per DSN than recommended, and there was a need for frequent office visits with the EAs, meaning that other patient groups had to wait. This created ethical challenges, forcing the DSNs to prioritise:

I don’t have time for that [i.e., frequent office visits with EAs], but I do it anyway. I fit them into time slots that don’t exist—it can be on my lunch break. It’s because I really want to help and they need it. (Participant 8)

They identified EAs who struggled with mental health and neuropsychiatric difficulties but lacked easy access to medical social workers or occupational therapists. They further desired education in mental healthcare, and more resources were needed to provide person‐centred care. They compared themselves with HCPs in paediatric care, who perceived them as having more time to spend on patients, describing this as a reason for delaying the transfer to adult care.

#### 3.3.3. Attempting to Implement Updated Contact Pathways

The DSNs aimed to improve contact pathways for EAs, recognising that traditional phone hours and delayed digital responses were inadequate, and noted the need for quick access to support but faced challenges with patient data privacy regulations that limited direct communication, such as through email. Nevertheless, some DSNs engaged with EAs via email or private social media despite not initiating contact. Flexibility was essential for EAs:

I feel like there’s a need for more spontaneity. Society is based on opportunities to go grocery shopping at 11 pm or seek healthcare whenever you need it. […] We absolutely don’t offer emergency healthcare services, but they can write on 1177 [i.e., a national digital health platform] whenever they want, even in the middle of the night. (Participant 1)

New strategies for engaging EAs include drop‐in office hours and after‐hours care. Digital visits were common and helpful, especially for those in remote areas or new cities. While adolescent‐friendly health services for EAs were suggested, referrals to these clinics could disrupt established care relationships.

## 4. Discussion

The aim of this study was to describe Swedish DSNs’ experiences of supporting EAs living with T1DM after the transfer from paediatric to adult care. The DSNs acknowledged emerging adulthood as a turbulent life period and the EAs as sensitive individuals, implying multilayered challenges for the DSNs and the provided support. The present results highlight the DSNs’ experiences of supporting EAs living with T1DM after the transfer.

The DSNs emphasised the individuals and relationships as most essential in diabetes care, with partnership being greatly emphasised in person‐centred care. This is in line with Ekman et al. [19], who presented routines using person‐centred care in clinical practice, including initiating partnerships, implementing partnerships, and maintaining partnerships by documenting the mutually agreed‐upon health plan. The studied DSNs had the ambition to optimise individual support for the EAs living with T1DM, but they experienced their hands as tied by organisational structural barriers, resulting in an inability to fully realise their ambition to provide person‐centred care. However, conflicts occurred in building trustful relationships in “uphill” situations after families had left paediatric care and established relationships. EAs faced a high risk of loss to follow‐up care [14]. The DSNs showed ambivalence about involving parents. However, Arnett [6] stated that EAs gradually take on adult responsibilities at their own pace, with DSNs noting that EAs are not seen as fully independent at 18, and parental involvement remains important in adult care.

The DSNs also focused on long‐term self‐management goals for EAs living with T1DM, recognising that EAs often struggled to fully meet the illness’s demands due to competing life priorities [7, 14, 20]. DSNs should therefore stress empowerment, without blaming EAs when treatment outcomes are suboptimal [7]. Malachowska et al. [10] described physical and psychological changes in EAs potentially leading to encounter new limitations that EAs living with T1DM were unaware of. These may impact functioning, well‐being, and how they perceive their illness. In addition, for many adolescents, the constant pressure often increases stress levels and decreases quality of life. The studied DSNs recognised their communication styles and reflected on their behaviours, emphasising the need to see EAs as capable individuals to enhance collaboration and focus on their resources and strengths [19]. The DSNs aimed to instil hope and help EAs reconcile with their situation, addressing the disease’s demands through enhanced existential support in self‐management [21–23].

The DSNs were struggling with obstacles to providing accessible care. The Swedish Agency for Health and Care Analysis [24] stated that Swedish healthcare services should strive for a systematic distribution of responsibilities between paediatric and adult care to facilitate more person‐centred transfers, although this has not yet been fully implemented. Joint transfer meetings are emphasised as positive in international guidelines [1, 14, 25, 26]. This study explores DSNs’ experiences in meetings and their feelings of vulnerability as new members in an established setting. Previous research also highlights the imbalance between healthcare resources and needs, along with frustration over heavy workloads [12, 27, 28]. From an international perspective, DSNs are often not included in standard diabetes care and, where they are, are typically undervalued and present in fewer numbers than recommended [11]. Ethical challenges in prioritisation derive from organisational issues, as DSNs often manage more patients than recommended. Additionally, EAs’ mental health and neuropsychiatric difficulties should ideally be addressed by an interdisciplinary diabetes team [7]. HCPs expressed frustration with the divide between healthcare specialties, including mental health teams, as noted in a study on supporting adults living with T1DM [27], linked to ethical challenges. The DSNs proposed improving communication systems and using familiar platforms for meetings to overcome obstacles in providing accessible care [29, 30]. Ekman et al. [19] stated that offering after‐hours and distance communication alternatives (e.g., chat functions and social media) are vital in person‐centred care.

### 4.1. Trustworthiness

Regarding credibility, a predetermined number of participants for inclusion cannot be established beforehand [16], so we intentionally recruited a diverse group of participants representing a wide range of ages, DSN work experiences, and hospitals. We determined that we had gathered sufficient variation in the experiences of DSNs supporting EAs living with T1DM when the codes from the last two interviews did not reveal any new subthemes. We considered elements that would enhance dependability. Members of the research team have experience working in diabetes teams in both paediatric and adult care (TW, JO, EL, and IL), bringing preunderstandings of the studied phenomena. Preunderstandings always involve risks and benefits, and texts can hold multiple meanings [15]. Nonetheless, we believe that the current results represent interpretations capturing the most probable meanings of the described experiences. During the decontextualisation phase, the codes were kept closely aligned with the transcribed text to mitigate the risk of overinterpretation [17], and experiences were also recognised. Since complex human experiences overlap, creating exclusive subthemes is not always feasible during the recontextualisation phase [15, 17]. Concerning transferability, we provided detailed descriptions of the participants and the procedures, but the evaluation of whether the results are transferable to other contexts rests with the readers [15]. The study’s reporting was assessed using the COREQ checklist for qualitative studies involving in‐depth interviews [31] (Appendix 1).

### 4.2. Limitations

All participants were female. Recruiting participants was difficult, potentially reflecting the organisational challenges experienced by DSNs. Regarding data collection, group interviews were initially planned, but due to recruitment difficulties and the DSNs’ opportunities to be interviewed during working hours, we decided to conduct individual interviews instead. Focus groups can capture group thinking, while individual interviews can gather more in‐depth experiences. It is possible that we could have obtained more diverse results if DSNs from different regions with diverse adult care and transfer routines had shared experiences in focus groups. A further limitation related to the data collection was that we did not pilot test the interview guide, and we did not ask the participants to reflect upon the interviewer’s performance. However, as previously mentioned in 2.4 Data Collection, we applied our standard procedure and evaluated the interview guide within the research team after the first interview, and before subsequent further use.

### 4.3. Implications for Practice

The findings of this study emphasise the importance of person‐centred care and the need for organisational support to help DSNs deliver optimal care for EAs living with T1DM. Addressing systemic barriers and incorporating mental health support within diabetes care are essential. More straightforward guidelines for parental involvement and enhanced communication systems can improve support. Continued training for DSNs and policy changes to enable a smoother transfer from paediatric to adult care are also crucial. These measures can greatly enhance the overall support and outcomes for EAs living with T1DM. In clinical practice, actionable recommendations are to ask for EAs’ permission to involve their parents when valued. When supporting EAs living with T1DM, we recommend HCPs to consider focusing on resources and strengths and instil hope as part of existential support. Concerning education in diabetes care, education in mental health and neuropsychiatric difficulties associated to living with T1DM should be emphasised.

## 5. Conclusions

The results provide insight into the multilayered experiences and challenges of DSNs supporting EAs living with T1DM after the transfer to adult care. DSNs have the ambition to optimise individual support but experience their hands as tied by organisational structural barriers. There was a highlighted need for education and training to support EAs living with T1DM that extends beyond self‐management to address life transitions and psychosocial challenges. Future supportive interventions should prioritise existential and psychosocial factors over standard medical treatment outcomes. While collaboration between paediatric and adult care exists, it lacks systematic national consensus or guidelines. Variations in transfer routines and ongoing care reported by DSNs indicate a need for further research.

## Conflicts of Interest

The authors declare no conflicts of interest.

## Funding

This work was supported by the Swedish Diabetes Foundation.

## Data Availability

The data that support the findings of this study are available from the corresponding author upon reasonable request.

## Appendix 1

**Table A1:** Consolidated criteria for reporting qualitative research (COREQ): a 32‐item checklist [1].

No.	Item	Guide questions/description	Text reference
*Domain 1: Research team and reflexivity* *Personal Characteristics*			
1.	Interviewer facilitator	Which author/s conducted the interviews or focus groups? Theresia Willman and Sara Olsson.	2.4 Data Collection
2.	Credentials	What were the researcher’s credentials? Sara Olsson, Paediatric nurse specialist, PhD‐student Theresia Willman, Paediatric nurse specialist Åsa Carlsund, Community health nurse, PhD, Associate professor Julia Otten, Medical doctor, PhD, Associate professor Madeleine Blusi, Registered nurse, Associate professor Elena Lundberg, Medical doctor, Paediatrics endocrinologist, PhD Ingela Lavin, Diabetes nurse specialist, Paediatric nurse specialist Åsa Hörnsten, R.N, Community health nurse, Professor	2.2 Researcher positionality
3.	Occupation	What was their occupation at the time of the study Read, 2. Credentials.	2.2 Researcher positionality
4.	Gender	Was the researcher male or female? All members were female.	2.2 Researcher positionality
5.	Experience and training	What experience or training did the researcher have? The interviewer TW had no previous experience in interviewing and the interviewer SO had experience from previously conducting interview studies. Other members of the research team have experiences of conducting qualitative research.	2.2 Researcher positionality

Relationship with participants			
6.	Relationship established	Was a relationship established prior to study commencement? The interviewers had no established relationship with the participants.	2.2 Researcher positionality
7.	Participant knowledge of the interviewer	What did the participants know about the researcher? e.g., personal goals, reasons for doing the research. The participants received information about the interviewers’ occupation, the research project, age, city of residence, and for interviewer so the preunderstandings of living with type 1 diabetes herself.	2.4 Data Collection, 2.6 Ethical Considerations, 4.1 Trustworthiness
8.	Interviewer characteristics	What characteristics were reported about the interviewer/facilitator? e.g., *preunderstanding, assumptions, reasons, and interests in the research topic.* The preunderstandings of the research team are reported.	2.4 Data Collection, 4.1 Trustworthiness

*Domain 2: Study design* *Theoretical framework*			
9.	Methodological orientation and Theory	What methodological orientation was stated to underpin the study? *e.g., grounded theory, discourse analysis, ethnography, phenomenology, qualitative content analysis*. Qualitative content analysis.	2.5 Data Analysis

*Participant selection*			
10.	Sampling	How were participants selected? *e.g., purposive, convenience, consecutive, snowball.* Purposive sampling.	2.3 Sampling and Recruitment
11.	Method of approach	How were participants approached? e*.g., face-to-face, telephone, mail, email*. E‐mail and face‐to‐face.	2.3 Sampling and Recruitment
12.	Sample size	How many participants were in the study? 10 participants.	2.3 Sampling and Recruitment
13.	Non‐participation	How many people refused to participate or dropped out? Reasons? Six Directors of departments at Swedish hospitals did not answered our e‐mails with information and letters of enquiry.	2.3 Sampling and Recruitment

Setting			
14.	Setting of data collection	Where was the data collected? e*.g., home, clinic, workplace*. Interviews were performed both digital and at diabetes specialist nurses’ workplaces.	2.4 Data Collection
15.	Presence of non‐participants	Was anyone else present besides the participants and researchers? No.	
16.	Description of sample	What are the important characteristics of the sample? *e.g., demographic data, date.* Participants’ workplace, age, gender, and work experiences in diabetes care.	2.3 Sampling and Recruitment

*Data collection*			
17.	Interview guide	Were questions, prompts, guides provided by the authors? Was it pilot tested? A semistructured interview with probing questions was used, but not pilot‐tested.	2.4 Data Collection
18.	Repeat interviews	Were repeat interviews carried out? If yes, how many? No.	
19.	Audio/visual recording	Did the research use audio or visual recording to collect the data? Both audio and visual recording.	2.4 Data Collection
20.	Field notes	Were field notes made during and/or after the interview or focus group? No.	
21.	Duration	What was the duration of the interviews or focus group? The interviews lasted from 16 to 52 min.	2.4 Data Collection
22.	Data saturation	Was data saturation discussed? Yes.	2.5 Data Analysis, 4.1 Trustworthiness
23.	Transcripts returned	Were transcripts returned to participants for comment and/or correction? No.	

*Domain 3: Analysis and findings* *Data analysis*			
24.	Number of data coders	How many data coders coded the data? Two authors (TW and SO) coded the transcribed interviews.	2.5 Data Analysis
25.	Description of the coding tree	Did authors provide a description of the coding tree? An overview of the results is presented in Table 1.	3 Results
26.	Derivation of themes	Were themes identified in advance or derived from the data? The themes were interpreted and abstracted from the data.	3 Results, 4 Discussions
27.	Software	What software, if applicable, was used to manage the data? No software was used.	
28.	Participant checking	Did participants provide feedback on the findings? No.	

*Reporting*			
29.	Quotations presented	Were participant quotations presented to illustrate the themes/findings? Was each quotation identified? e*.g., participant number.* Quotations are presented together with participant numbers.	3 Results
30.	Data and findings consistent	Was there consistency between the data presented and the findings? Yes.	3 Results, 4 Discussions, 4.1 Trustworthiness
31.	Clarity of major themes	Were major themes clearly presented in the findings? Yes, a main theme, three themes, and 10 subthemes were presented in the results.	3 Results
32.	Clarity of minor themes	Is there a description of diverse cases or discussion of minor themes? Yes.	3 Results, 4 Discussions, 4.1 Trustworthiness
